# Support Vector Machine Optimized by Genetic Algorithm for Data Analysis of Near-Infrared Spectroscopy Sensors

**DOI:** 10.3390/s18103222

**Published:** 2018-09-25

**Authors:** Di Wang, Lin Xie, Simon X. Yang, Fengchun Tian

**Affiliations:** 1 College of Communications Engineering, Chongqing University, Chongqing 400044, China; diwang871106@gmail.com (D.W.); FengchunTian@cqu.edu.cn (F.T.); 2 School of Engineering, University of Guelph, Guelph, ON N1G 2W1, Canada; xielingtoefl@gmail.com

**Keywords:** support vector machine, NIR sensor, feature selection, genetic algorithm, cultivation region discrimination

## Abstract

Near-infrared (NIR) spectral sensors deliver the spectral response of the light absorbed by materials for quantification, qualification or identification. Spectral analysis technology based on the NIR sensor has been a useful tool for complex information processing and high precision identification in the tobacco industry. In this paper, a novel method based on the support vector machine (SVM) is proposed to discriminate the tobacco cultivation region using the near-infrared (NIR) sensors, where the genetic algorithm (GA) is employed for input subset selection to identify the effective principal components (PCs) for the SVM model. With the same number of PCs as the inputs to the SVM model, a number of comparative experiments were conducted between the effective PCs selected by GA and the PCs orderly starting from the first one. The model performance was evaluated in terms of prediction accuracy and four parameters of assessment criteria (true positive rate, true negative rate, positive predictive value and F1 score). From the results, it is interesting to find that some PCs with less information may contribute more to the cultivation regions and are considered as more effective PCs, and the SVM model with the effective PCs selected by GA has a superior discrimination capacity. The proposed GA-SVM model can effectively learn the relationship between tobacco cultivation regions and tobacco NIR sensor data.

## 1. Introduction

The quality of tobacco is affected highly by the cultivation environment, such as the temperature, rainfall, soil and so on. Generally, tobaccos from the same region usually have the similar characteristic and fragrance style. Meanwhile, cigarettes are ranked according to the fragrance styles and the retail price is set accordingly. However, tobacco leaves from different regions may be blended together during the acquisition and shipping process intentionally or unintentionally, which makes inspection difficult before being put into production. As the manual identification of the growing region is not reliable and cannot meet the requirement, an automatic and intelligent identification approach for the tobacco growing regions is highly desirable.

With the development of spectral sensor technology, the application of the near-infrared (NIR) sensor has been widely used in many fields [1,2,3,4,5,6]. The spectra obtained from the NIR sensor have the potential to extract corresponding feature information for the samples. In recent years, the NIR spectral sensor technology has developed quickly as a powerful analytical method in the tobacco industry and proven its effectiveness for both qualitative and quantitative analyses due to having the characteristics of rapidity, simplicity and non-destructive measurements [7,8,9], especially when it is applied to tobacco classification problems [8,10,11,12].

A lot of research works have been conducted on the classification of tobacco cultivation regions with different algorithms using near-infrared (NIR) sensors. Zhu, Gong, Li, and Yu [13] identified the cultivation regions of tobacco with the high dimensional feature grouping method, which means they sorted all NIR spectra features according to importance scores of the features from small to large and then divided them into twelve groups, and they made the feature selection to get the optimal feature subset with different feature groups by calculating the error rate. Zhang, He, and Ye [14] proposed the least square support vector machines (LS-SVM) to determine the tobacco producing area using the NIR sensor with the wavelength range from 1101 to 2395 nm. In their research, 4 to 12 principal components (PCs) were examined separately as the inputs of LS-SVM models and results showed that the model with 12 PCs obtained the larger correlation coefficient and smaller root mean square error. Maha Hana, McClure, Whitaker, White, and Bahler [15] employed artificial neural networks (ANNs) with 19 points of the NIR spectra based on the multiple sensors to classify whether the burley tobacco grows in the USA or outside the USA, and obtained high prediction accuracy. Ni et al. [10] applied improved and simplified K-nearest neighbors classification algorithm (IS-KNN) to discriminate the cultivation areas of more than 1000 Chinese flue-cured tobacco leaf samples based on the spectral sensors and they used one original method to optimize the number of significant PCs based oil analysis of error and cross-validation. It can be observed from the above studies that selecting PCs for most models is just according to the scores of the PCs.

Support vector machine (SVM), firstly proposed by Vapnik, has been proven to be a powerful technique for pattern recognition, classification, and regression in many fields [16,17,18,19,20,21,22,23]. It has attracted a lot of attention due to its remarkable advantages: (1) effective in high dimensional spaces; (2) suitable for small samples set; (3) reasonable mathematic support; and (4) efficient perform as a non-linear classifier. In recent decades, SVM has been applied widely and successfully in solving classification problems, such as recognizing bowel sound [24], classifying vegetable pests [25], identifying alcohol consumption [26]. There are also some applications of SVM in the tobacco industry, such as classifying fragrant styles and evaluating the aromatic quality of flue-cured tobacco leaves [27], classifying the producing year of tobacco [12] and tobacco leaf grades [28]. In our early study on discrimination of tobacco growing regions based on NIR data, it was found that the SVM model is a suitable classifier [29].

In this study, the genetic algorithm (GA) was introduced to improve the performance of the SVM classifier. Principal component analysis (PCA) was used to extract the features from the de-noised NIR sensor data of tobacco and obtained principal components (PCs) corresponding to the score range from small to large. The first 25 PCs from PCA was chosen as the original inputs of the SVM model, and a set of the trial number of PCs from the 25 ones was examined on training to find out the most proper number of PCs for a model establishment. The input subsets with those corresponding number of PCs were tried on evaluating the performance of the SVM model and compared how many PCs can optimize the SVM classifier to the maximum extent. During the process of selecting PCs in different input subsets, GA was proposed to find the most effective PCs for the corresponding input subsets. In order to prove the availability of the proposed approach, the selection of parameters and kernel function for the SVM model were discussed in details for performance improvement. The experimental results were explained clearly with the evaluation parameters by figures and tables. This paper is based on the previous work in Reference [30].

## 2. Materials and Methods

**Figure 1:**
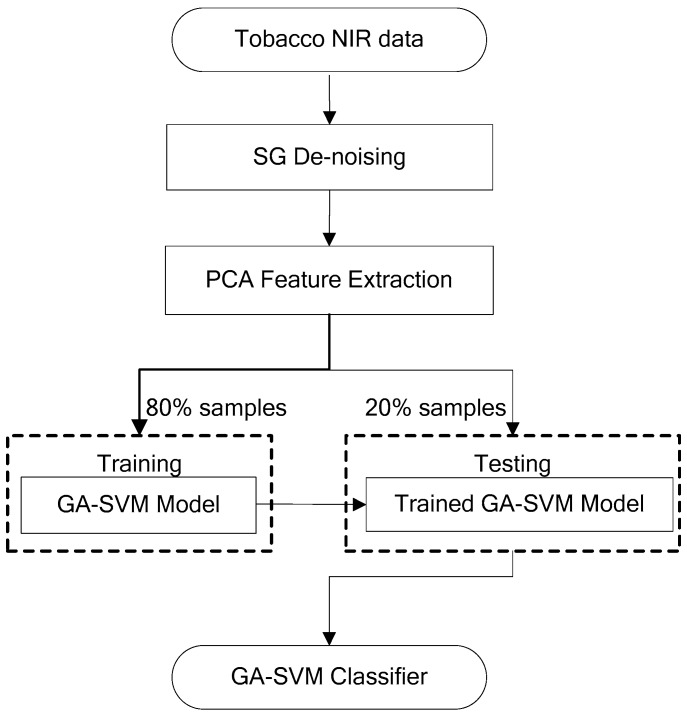
Framework of tobacco cultivation region classifier.

The framework of this study is shown in Figure 1. Firstly, the Savitzky–Golay (SG) de-noising method was applied to eliminate the noise in the NIR spectra data, and PCA technology was used to extract the main features and compress the dimension of de-noised NIR spectral sensor data. Then, all dataset was divided into the training set (80%) and testing set (20%) randomly, and the former was used for optimal feature selection by GA to get the optimal input subset for establishing the SVM classifier. After that, the testing set was used for testing the performance of the SVM model.

### 2.1. Tobacco Database

**Figure 2:**
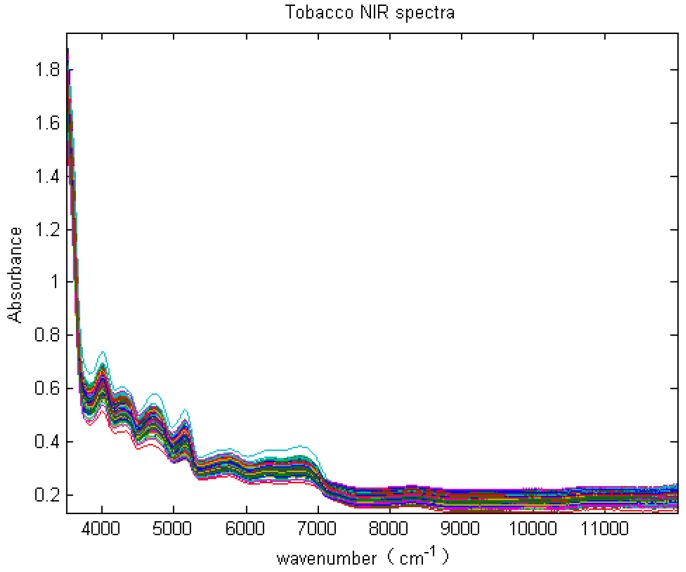
Raw NIR spectra of the 332 samples.

A total of 332 tobacco samples were collected from four different regions in Guizhou Province by the Guizhou Tobacco Science Research Institute of China. Due to the suitable climate and soil condition in the local regions, tobacco cultivation is very popular there. However, it is very hard to discriminate the tobaccos from the regions. The number of tobacco samples collected from the four regions is given in Table 1. The NIR spectra of the 332 samples were recorded with Thermo Antaris 2 with multiple sensors (Thermo Fisher Scientific Inc., Waltham, MA, USA). The spectra are with the resolution of 8 cm^−1^ and 64 scans. The NIR range is from 3499 cm^−1^ to 12,000 cm^−1^, which is shown in Figure 2. It shows that there are significant fluctuations from 3500 cm^−1^ nm to 7000 cm^−1^ and the spectra have peaks at 4004 cm^−1^, 4313 cm^−1^, 4727 cm^−1^, and 5163 cm^−1^, which are frequency doubling and sum-frequency absorption of hydrogen containing groups such as C–H, O–H, N–H, and S–H. Generally, the near-infrared band of sum frequency is located between 4000 cm^−1^ and 5000 cm^−1^, while the first order harmonic and second harmonic are ranged from 5556 cm^−1^ to 7143 cm^−1^, and the third and fourth or higher harmonic focus on the band between 11,111 cm^−1^ and 12,800 cm^−1^.

### 2.2. De-Noising for the Raw Spectra of the Samples

The NIR spectra gathered with the NIR sensor are high-dimensional. The value of absorbance is recorded at total 2084 points, so the dimension of the samples is 2084 in this study. As the spectra carry the internal information of the atomic bond of the molecule, it is very effective to do the qualitative and quantitative analysis for them.

However, unexpected noise from the NIR spectral sensor data and human operation will be introduced during the NIR sensor data acquisition. The noise may degrade the signal to noise ratio (SNR) and affect the model performance, so de-noising raw NIR spectral sensor data is very significant in the model establishment.

The SG filter is applied for the raw NIR data to eliminate the noise in the NIR spectral sensor data. The principle of the de-noising lies in replacing the contaminated NIR data with the average value that calculated from the contaminated data. A quadratic polynomial is adopted and the size of the sliding window is set to 121 in this study.

### 2.3. Outlier Identification and Feature Extraction

In this study, Mahalanobis distance was used to detect the outliers. The average values of all the spectra of the samples were firstly calculated and used to build an average spectrum. Then the Mahalanobis distance between each spectrum and the average spectrum was calculated, and was made as the outlier measurement of each spectrum. The spectrum with Mahalanobis distance from the average spectrum bigger than the threshold would be considered as one outlier. All the outliers were eliminated.

**Figure 3:**
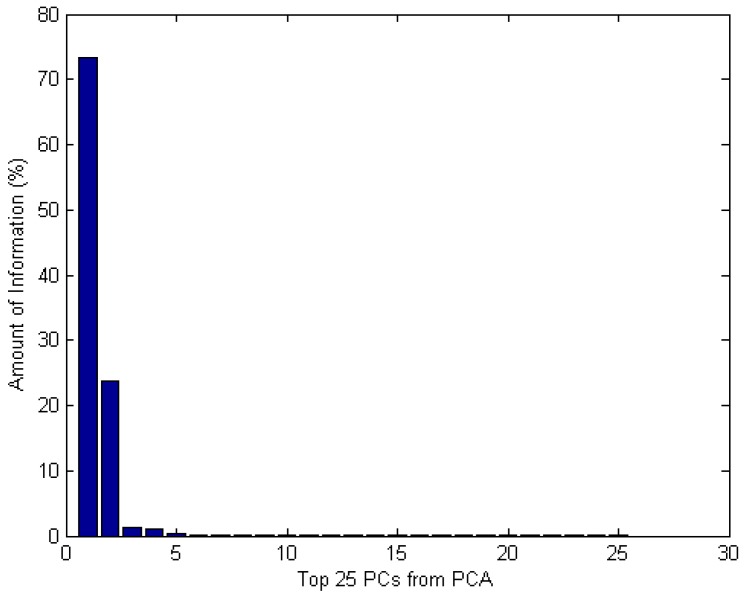
Amount of information for each of the top 25 PCs from PCA.

PCA is one of the most popular approaches to extract features from high dimensional data. It was applied for the de-noised NIR spectral sensor data with SG smoothing method. In order to keep as much information as possible, the full spectral range of the raw data from 12,000 cm^−1^ to 3499 cm^−1^ was adopted. There were total 2084 data points in each tobacco NIR spectrum, which tends to over-fitting easily due to the high dimension, so PCA was applied to extract the main features from the de-noised NIR spectral sensor data, which is also called reducing dimension. Each data firstly had the mean value of the corresponding columns subtracted and the covariance matrix of the new dataset was calculated. The eigenvalues and eigenvectors matrix can be calculated from the covariance matrix and the PCs from the PCA are ranked according to the value of the eigenvalues from large to small, so the PCs in the eigenvectors with larger eigenvalues contained more useful information or energy of the dataset. The number of the principal components is very significant, as too much noise and other redundant information will be carried into the data if it is too big but useful information will be lost if it is too small. Generally, a certain number of PCs are chosen simply from front to back, which means the selection is just according to the value of their eigenvalues, however, although such PCs carry the most useful information of the data, some of them may be not effective for the model to be built. In this study, the top 25 PCs from PCA algorithm were extracted as the original input for the model, as they almost occupied 100% of the information of the data, which was shown in Figure 3.

It is very important to select the number of the PCs, since too many PCs may contain too much noise and too few PCs may lose the useful information. In this study, a series trial number of PCs (6, 8, 10, 12, 14, 16) from the 25 PCs were combined to be different input subsets for the model establishment and the most suitable input subset was selected based on the prediction accuracy of the SVM model.

### 2.4. Genetic Algorithm Optimized Support Vector Machine Approach

**Figure 4:**
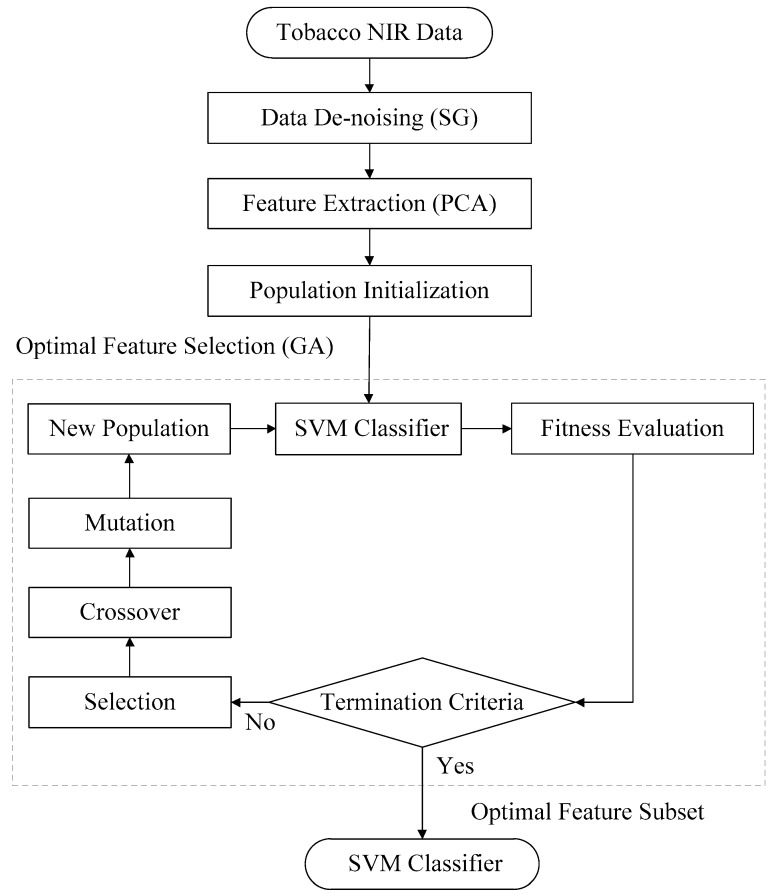
Flow diagram of the proposed GA-SVM classifier.

In order to feed the more effective features into the SVM model, GA was proposed to select the most model-effective PCs for the corresponding input subsets. The detailed procedure of the GA-SVM is shown in Figure 4. Firstly, the population initialization is set randomly in the first 25 PCs from the PCA algorithm. Then the optimal feature selection with GA is followed as shown in the dashed part. The prediction accuracy of the SVM classifier is used for measurement of the fitness value for each individual. The fitness value biased roulette method is used to select the suitable parents. The input subset with a larger fitness value is more likely to be selected as parents. All parents carry more effective genetic information for the SVM model. Parents are mated randomly and reproduction carries on here. Crossover and mutation, as the two main genetic operations, are functioned to produce the offspring and boost the new population. One-point crossover and uniform mutation are adopted in this study. After several generations, genes with a higher fitness value are passed on and inherited. The evolution continues until meeting the termination criteria. Finally, the optimal input subset with the best fitness is selected for the SVM classifier.

### 2.5. Support Vector Machine Algorithm

Although the SVM was originally designed for binary pattern recognition problems, it has been extended to solve multi-class problems. Given (x,y) is a set of samples, $\vec{x}$ is the vector of NIR spectral sensor data, and y = {−1,1}, which is in the case of two classes, and the linear decision hyperplane is given as

(1) $$g\left(\vec{x}\right)=\vec{w}\cdot \vec{x}+b,$$

where $\vec{w}$ is the weight vector and *b* is the bias, and · is the inner product operator. The separating hyperplane is $g\left(\vec{x}\right)=0$ and the corresponding classifier is set to be sign of $g\left(\vec{x}\right)$. The Euclidean distance between any ${\vec{x}}_{i}$ to the separating hyperplane is given by

(2) $$d=\frac{1}{\left\|w\right\|}\left|g\left({\vec{x}}_{i}\right)\right|,$$

where $\left\|\vec{w}\right\|$ is the L2 norm. The maximize margin between $g\left(\vec{x}\right)=1$ and $g\left(\vec{x}\right)=-1$ is equal to minimize $\frac{1}{2}\|\vec{w}{\|}^{2}$. In order to improve the capacity of the model, a slack variable *ε* is introduced to boost the tolerance of the distance.

The goal of the classification is to find the optimal separating hyperplane (OSH) which is equal to solve the following quadratic equation,

(3) $$\underset{w,b,\varepsilon }{\min}\frac{1}{2}\|\vec{w}{\|}^{2}+C\sum_{i=1}^{N}\varepsilon_{i},$$

$$\mathrm{subject}\;\mathrm{to}\;y_{i}\left[\vec{w}\cdot \vec{x}+b\right]\geq 1-\varepsilon_{i}$$

where *i* is the sample number, *C* is the penalty constant, $\varepsilon_{i}$ is the slack variable. The *w* and *b* can be searched by solving the following equation with Lagrange multipliers method,

(4) $$\underset{\alpha_{i}}{\min}\frac{1}{2}\sum_{i=1}^{N}\sum_{j=1}^{N}\alpha_{i}\alpha_{j}y_{i}y_{j}K\left({\vec{x}}_{i}\cdot {\vec{x}}_{j}\right)-\sum_{i=1}^{N}\alpha_{i},$$

$$\mathrm{subject}\;\mathrm{to}\;\sum_{i=1}^{N}\alpha_{i}{\vec{y}}_{i}=0,\;0\leq \alpha_{i}\leq C\;\left(i=1,\ldots N\right)$$

where *K*(${\vec{x}}_{i}\cdot {\vec{x}}_{j}$) is the kernel function and plays a significant role in the SVM model. Normally, there are four kernel functions: Linear function, polynomial function, radial basis function (RBF) and sigmoid function [30]. In this study, RBF was selected as the kernel function, and its function is given as,

(5) $$K\left({\vec{x}}_{i}\cdot {\vec{x}}_{j}\right)=-\frac{\|{\vec{x}}_{i}-{\vec{x}}_{j}{\|}^{2}}{2\sigma^{2}}.$$

Although the basic SVM is applicable to two classes, the method of SVM for multi-classification is to adopt the idea of the decision tree. It starts from the root node, divides the category contained by the node into two subclasses, and then further divides the two subclasses, and so on, until only one class is included in the subclasses. Thus one inverted binary tree is obtained in this way and the SVM classifier is trained on each decision node of binary tree to classify the samples with multi-classes.

### 2.6. Model Evaluation

The prediction accuracy is the significant parameter to evaluate the overall performance in the classification of the tobacco cultivation region and it is defined as

(6) $$P_{a}=\frac{n_{r}}{N_{t}}\;$$

where $n_{r}$ is the number of samples predicted rightly and $N_{t}$ is the number of samples for prediction. In this study, $N_{t}$ is set to be 266 and 66 in the training and testing stages, respectively.

One two-by-two confusion matrix showed in Table 2 supports the evaluation criteria for the models. As shown in Table 2, *n* means the number of samples, and the parameters are defined according to the styles of the given label and the predicted label, where $n_{TP}$ is the number of positive samples that are labeled as positive, $n_{TN}$ is the number of negative samples labeled as negative, $n_{FP}$ is the number of positive samples labeled as negative, and $n_{FN}$ is the number of negative samples labeled as positive [30]. The functions of the evaluation criteria are given as

(7) $$\gamma_{TP}=\frac{n_{TP}}{n_{TP}+n_{FN}}\;$$

(8) $$\;\gamma_{TP}=\frac{n_{TP}}{n_{TP}+n_{FN}}\;$$

(9) $$\;\gamma_{PP}=\frac{n_{TP}}{n_{TP}+n_{FP}}\;$$

(10) $$\;\gamma =\frac{2n_{TP}}{2n_{TP}+n_{FP}+n_{FN}}\;$$

where $\gamma_{TP}$ is sensitivity rate, which is a measure of the ability to detect the positive patterns; $\gamma_{TN}$ is specificity rate, which is means the ability to specify the negative patterns; $\gamma_{PP}$ is the precision rate, which represents the ability to predict the positive patterns; and γ is F1-score, which considers both the precision and sensitivity of the test.

## 3. Results and Discussion

In order to investigate how the GA optimizes the SVM model, different trial values were tested and discussed, the procedure of the feature selection with GA was represented in details in this section. All simulations are implemented on a Windows 10 operating system using MATLAB R2014a, which is running on a laptop with Intel (R) Core (TM) i5-4210U CPU 1.70 GHz and 2.40 GHz RAM. The machine learning analysis is conducted mainly using the MATLAB toolboxes.

### 3.1. Selection of Parameters

A grid search method and 5-fold cross-validation approach was adopted to select the parameter pair (*C*, *σ*) for SVM model. Six input subsets with the different fixed number of first PCs (6, 8, 10, 12, 14, 16) from PCA were used for the input of the SVM model. The result was shown in Table 3. As shown in the Table, when the first 14 PCs were selected for the input of the model, the best prediction accuracy for 5-fold cross-validation was obtained as 78.6% with the parameter pair *C* = 2 and *σ* = 2. The search time for 14 inputs was 24 s and for other inputs were all around 24 s. However, the computation time was slightly longer as the number of inputs increased. Therefore, the parameter pair was selected as 2 and 2 respectively in this study.

### 3.2. Feature Extraction with GA

Population size and genetic operators are two significant parameters for GA, and they are discussed in detail as follows.

#### 3.2.1. Population Size

**Figure 5:**
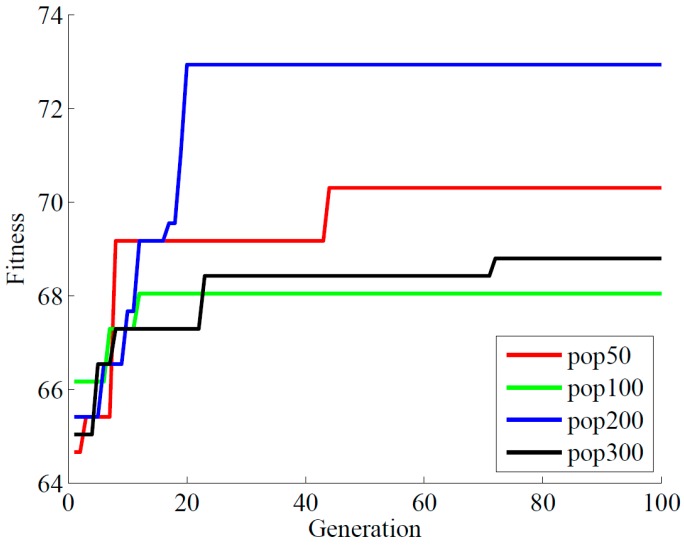
The selection of population size for the GA-SVM model [30].

The population size in GA is a very important parameter, as it has impact on the search speed and convergence rate. It will make the search too complicated if it is too large, while it will stop the search too early and miss some good solutions if it is too small. Thus different population sizes were examined to find the most appropriate one. The best fitness value with different population size is shown in Figure 5. It can be found that the larger the population is, the more quickly the optimal solution achieved. Meanwhile, the larger population size will need more generations to achieve the fitness. However, a too large population size is likely to add to the complexity of the problem and decrease reliability. So the appropriate population size was selected to be 200 in this study.

#### 3.2.2. Genetic Operators

**Figure 6:**
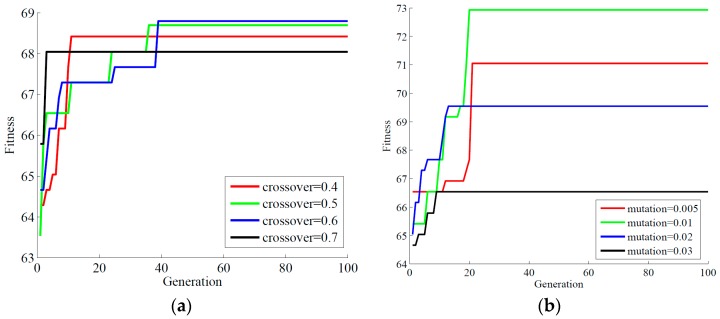
The selection of crossover rate and mutation rate for the GA-SVM model [30]. (**a**) Crossover rate; (**b**) Mutation rate.

The genetic operators include crossover and mutation in GA, which determines the diversity of genes in GA. The probabilities of crossover and mutation play a significant role in the convergence speed to achieve the fitness. Generally, the range of the probability of crossover is usually set from 0.5 to 1, and that of a probability of the mutation is likely to set at less than 0.1. The biased roulette wheel method was used to select parents and one-point crossover was adopted to produce more superior offspring for the GA-SVM model. In order to find the most appropriate value for the probabilities of crossover and mutation, a series of trial values were tested and the result was shown in Figure 6. The fitness value keeps on going up when the crossover rate increases from 0.4 to 0.6, but drops down when the crossover rate is over 0.6, which means that high crossover rate can reproduce more offspring to achieve more chance of good solution, but too big crossover rate will degrade performance. Therefore, 0.6 was selected as the crossover rate. It can be found that the fitness value rises when the mutation rate increases from 0.005 to 0.01 but drops down when the mutation rate is over 0.01. It indicates that the model will lose a good solution when the mutation rate is too big. Therefore, 0.01 was selected to be the mutation rate in this study.

#### 3.2.3. Selection the Number of PCs with GA

The sensitivity of the PCs to the discrimination of tobacco cultivation region was examined by GA with the population size of 200, the maximum generation of 100, the probability of crossover of 60% and the probability of mutation of 1%. The first 25 PCs with larger eigenvalues (more information) were selected by PCA as the original inputs. A total of six different input subsets were chosen, where the number of PCs in these six input subsets was 6, 8, 10, 12, 14 and 16. The PCs in the input subset were selected from the first 25 PCs by the GA, and the selected one was labeled with 1 and the unselected one labeled with 0, as shown in Table 4. Very interestingly, it is observed that the selection of PCs by the GA is not according to the size of the eigenvalues, but depends on the sensitivity to the discrimination of tobacco growing regions. The 1st, 6th, 7th, 8th, 10th and 11th PCs were selected regardless of how many input numbers were chosen, which showed that they were effective PCs for the discrimination problem of tobacco growing regions. In addition, as the number of inputs increases, the selection of effective PCs depends on their interaction rather than how much information the PCs possess.

In order to make a comparison with the SVM model, the prediction accuracy (*P_a_*) of the GA-SVM model was shown in Table 5 as well as that of the SVM model with the first corresponding PCs. It can be observed that the GA-SVM model performs better for almost every input subset. However, for both of the two models, the prediction accuracy keeps going up as the input number increases and achieves the highest value when 14 inputs are chosen, then it drops down as the input number are more than 14. The prediction accuracy for the GA-SVM model is up to 83.3% when 14 PCs were selected by GA, which was 3% higher than that of SVM. Meanwhile, for the same number of inputs, the input subset of GA-SVM model possessed less information from the raw NIR spectra than that of SVM model, but it had better performance. It indicated again that GA selected the inputs for the SVM model to classify the cultivation regions of tobacco mainly based on the sensitivity of the PCs but not the information the PCs has.

### 3.3. The Evaluation of the Proposed Model

When 14 PCs were selected as the input by GA, the comprehensive assessment for cultivation region classifiers was examined in terms of $\gamma_{TP}$, $\gamma_{TN}$, and γ, the result for each class was depicted in Table 6. It can be observed that GA-SVM model had a better performance than the SVM model in distributing the tobacco leaves from north part and southwest part of Guizhou Province. Both $\gamma_{TP}$ and γ for the GA-SVM model in class 1 (0.92 and 0.96, respectively) and class 4 (0.88 and 0.94, respectively) were larger than that for the SVM model ($\gamma_{TP}$ is 0.67 and 0.82 in class 1, γ is 0.76 and 0.9 in class 4, respectively). In class 1, they were 0.25 and 0.2 higher, respectively, than the latter model. Values of the four evaluation criteria for the two models were almost the same in class 2 and all were around 0.8, and it shows that both of two models have the same performance for the tobacco leaves from the middle of the province. The performance of GA-SVM model was slightly inferior to the SVM model for the tobacco leaves from northwest of Guizhou Province. With the exception of $\gamma_{TN}$, the value of the other three evaluation criteria was very small (0.67) for the GA-SVM model in class 3, which was about 0.09 lower than that of the SVM model. Considering the overall assessment, the GA-SVM model had a better capacity than the SVM model to classify tobacco from different cultivation regions, so it had a superior application.

In comparison with those previous works that used different models for tobacco classification problems based on NIR spectra, although the accuracy of the GA-SVM in this study (0.83) is a little inferior to the results in some other previous works [11,27] (about 0.90), the result is superior to the SVM model (0.80), so it demonstrates that the GA can select the effective PC input subset for the SVM model, which is the primary purpose of the study. The sensitivity rate and specificity rate of the GA-SVM model (0.92 and 1.00, respectively) for class 1 are much higher than that achieved by Montano-Moreno et al. [31] (0.91 and 0.74, respectively). The best prediction rate of GA-SVM (1.00) is bigger than that obtained by Besalu et al. [32] (0.865).

## 4. Conclusions

In order to investigate the effective PCs from PCA for the tobacco cultivation-region classification, in this study the first 25 PCs from PCA were firstly chosen as the original input, and six input subsets with different number of PCs (6, 8, 10, 12, 14, 16) from the 25 ones were then examined for the SVM model. The genetic algorithm was proposed to select the most effective PCs for the corresponding input subsets. The setting parameters of GA were discussed in detail to find the most suitable GA parameters. A series of experiments were conducted with and without the GA. Interestingly it was found that the more effective PCs may not be the PCs that have more information in general. Comparative studies show that the SVM classifier with the optimal PCs selected by GA has a superior performance with the SVM classifier with same number of PCs from the first component. The results demonstrated the tobacco cultivation region classifier relied on the sensitivity of the PCs but not the information they possess from the raw tobacco NIR spectral sensor data, and the GA is a feasible method for feature selection in classification problems.

## Figures and Tables

**Table 1:** The number of tobacco samples collected in four different regions.

Class 1 (North)	Class 2 (Middle)	Class 3 (Northwest)	Class 4 (Southwest)	Total
38	144	70	80	332

**Table 2:** The confusion matrix.

		Predicted Label	
		Positive	Negative
**Given Label**	Positive	$n_{TP}$	$n_{FN}$
	Negative	$n_{FP}$	$n_{TN}$

**Table 3:** Parameters selection with grid search method and 5-fold cross-validation. *n*: the number of PCs in corresponding input subset; *C*: the penalty constant; *σ*: the width in RBF; *P_cva_*: the best prediction accuracy of 5-fold cross-validation; *t*: time consumption.

*n*	*C*	*σ*	*P_cva_* (%)	*t* (s)
6	2.0	2.0	59	20
8	1.4	2.0	66.2	21
10	2.0	1.4	72.2	23
12	2.0	1.4	75.6	24
14	2.0	2.0	78.6	24
16	2.0	0.7	75.6	28

**Table 4:** Optimal individual PCs selected by GA based on SG smoothing of the NIR spectral sensor data. *N_i_*: the *i*-th number of PC in PCA.

	Input Number					
*N_i_*	6	8	10	12	14	16
1	1	1	1	1	1	1
2	0	0	0	0	1	1
3	0	1	1	1	1	1
4	0	0	0	0	0	1
5	0	0	1	0	1	1
6	1	1	1	1	1	1
7	1	1	1	1	1	1
8	1	1	1	1	1	1
9	0	0	1	1	1	0
10	1	1	1	1	1	1
11	1	1	1	1	1	1
12	0	0	1	1	1	1
13	0	0	0	0	1	1
14	0	0	0	0	0	1
15	0	0	0	1	1	0
16	0	0	0	0	0	0
17	0	0	0	0	0	0
18	0	0	0	0	0	0
19	0	0	0	0	0	0
20	0	0	0	0	0	1
21	0	1	0	1	1	1
22	0	0	0	0	0	0
23	0	0	0	1	0	0
24	0	0	0	0	0	1
25	0	0	0	0	0	0

**Table 5:** Prediction accuracy of GA-SVM with PCs from GA and SVM model with the first corresponding number of PCs. P_a_: prediction accuracy; *I_m_*: the amount of information.

	GA-SVM		SVM	
Input Number	*P_a_* (%)	*I_m_* (%)	*P_a_* (%)	*I_m_* (%)
6	72.7	72.4	60.6	99.6
8	75.8	74.1	67.0	99.8
10	75.8	74.7	68.2	99.8
12	74.2	74.2	77.3	99.8
14	83.3	98.8	80.3	99.9
16	78.8	99.8	78.8	99.9

**Table 6:** The result of two models in terms of four evaluation parameters on testing set. $\gamma_{TP}$: sensitivity rate; $\gamma_{TN}$: specificity rate; $\gamma_{PP}$: precision rate; γ: F1-score [30].

	**Class 1: North**				**Class 2: Middle**			
	$\gamma_{TP}$	$\gamma_{TN}$	$\gamma_{PP}$	***γ***	$\gamma_{TP}$	$\gamma_{TN}$	$\gamma_{PP}$	***γ***
**GA-SVM**	0.92	1	1	0.96	0.84	0.83	0.75	0.79
**SVM**	0.67	0.98	1	0.76	0.88	0.78	0.75	0.79
	**Class 3: Northwest**				**Class 4: Southwest**			
	$\gamma_{TP}$	$\gamma_{TN}$	$\gamma_{PP}$	***γ***	$\gamma_{TP}$	$\gamma_{TN}$	$\gamma_{PP}$	***γ***
**GA-SVM**	0.67	0.93	0.67	0.67	0.88	1	1	0.94
**SVM**	0.75	0.94	0.71	0.75	0.82	1	0.89	0.9
